# A systematic review and meta-analysis of seroprevalence surveys of ebolavirus infection

**DOI:** 10.1038/sdata.2016.133

**Published:** 2017-01-31

**Authors:** Hilary Bower, Judith R. Glynn

**Affiliations:** 1 Department of Infectious Disease Epidemiology, London School of Hygiene & Tropical Medicine, London WC1E 7HT, UK

**Keywords:** Viral infection, Diagnostic markers

## Abstract

Asymptomatic ebolavirus infection could greatly influence transmission dynamics, but there is little consensus on how frequently it occurs or even if it exists. This paper summarises the available evidence on seroprevalence of Ebola, Sudan and Bundibugyo virus IgG in people without known ebolavirus disease. Through systematic review, we identified 51 studies with seroprevalence results in sera collected from 1961 to 2016. We tabulated findings by study population, contact, assay, antigen and positivity threshold used, and present seroprevalence point estimates and 95% confidence intervals. We classified sampled populations in three groups: those with household or known case-contact; those living in outbreak or epidemic areas but without reported case-contact; and those living in areas with no recorded cases of ebolavirus disease. We performed meta-analysis only in the known case-contact group since this is the only group with comparable exposures between studies. Eight contact studies fitted our inclusion criteria, giving an overall estimate of seroprevalence in contacts with no reported symptoms of 3.3% (95% CI 2.4–4.4, *P*<0.001), but with substantial heterogeneity.

## Introduction

Knowing if ebolavirus infection manifests asymptomatically is critical to understanding its spread and to estimating the role herd immunity could have in reducing transmission. Investigating unrecognised infections could also help in the development and targeting of vaccines. However, despite a surprisingly large number of investigations into the seroprevalence of ebolavirus IgG since the first outbreak in Yambuku, Zaire (now Democratic Republic of Congo)^1–51^, consensus on results has proved elusive. The main reasons for this are the range of findings, positive results in unexpected locations, and a lack of confidence in immunofluorescence antibody (IFA) tests used in early studies.

Concerns about IFA specificity stem largely from studies showing positive results in populations expected to be negative, although the most frequently cited—in 200 Panamanian Indians with no known exposure—found only one Ebola virus IgG positive on a high cut-off giving a specificity of 99.5%^4^. Unexpected seropositivity has also been seen in African countries without reported cases of ebolavirus disease (EVD) such as the Central African Republic, Cameroon and Zimbabwe, only some of which can be attributed to using low test cut-offs. But, as some ELISA-based studies have produced similar findings^37,38^, these positive results may indicate zoonotic exposure with filoviruses or unrecognised human-to-human transmission rather than poor specificity.

‘Asymptomatic’ status can only be defined for a certain period, such as during an outbreak, though excluding mild symptoms is difficult. In outbreak areas asymptomatic subjects could have experienced unrecognised symptomatic EVD in the past so, even apart from problems with the test, ebolavirus antibody seropositivity does not necessarily mean asymptomatic infection.

We aimed to provide an up-to-date and easily accessible overview of serological findings to date, to help researchers contextualise studies prompted by the 2014–16 West Africa epidemic. The most comprehensive review of ebolavirus serology—Kuhn’s Filoviruses: A Compendium of 40 years of Epidemiological, Clinical and Laboratory Studies^52^—covers work to 2008. In addition to reviewing this key reference, we carried out a systematic review of serosurveys in people without symptoms of EVD up to July 2016.

## Results

### Characterisation of seroprevalence surveys of IgG antibodies to ebolavirus

We identified 51 studies covering 84 sample populations reported to have had no symptoms of EVD during the outbreak period, or to have come from populations with no known outbreaks. In total these studies investigated the presence of ebolavirus IgG in 44,147 subjects using samples collected since 1961.

Thirteen studies reported 16 study populations involving 2,664 participants with household or known case-contact^5–7,9,12,36,41,42,45,47,49–51^. Eleven studies reported 17 study populations covering 5,327 participants living in outbreak areas but without reported case-contact^5–7,9,14,33,39,40,42,43,46^. The remaining studies reported on 51 groups involving 36,156 subjects from general populations, often in settings ecologically similar to ebolavirus outbreak areas but without known cases of EVD^1–3,5,8,10,11,13,15–35,37,38,44–46,48,51^.

Table 1 (available online only) gives a detailed breakdown of the study populations, test methods and results.

### Overall estimates of ebolavirus seroprevalence in asymptomatic individuals

Only the group with known case-contact had exposures that are comparable across studies and are therefore appropriate to combine by meta-analysis. In this group eight study populations fulfilled the inclusion criteria of testing by ELISA or using a IFA cut-off ≥1:64 (ref. 5,^36^,^41^,^42^,^47^,^49–51^). Pooling these results gave an overall estimate of seroprevalence in asymptomatic people with known case-contact of 3.3% (95% CI 2.4–4.4, *P*<0.001), but with substantial heterogeneity due to three small studies with higher estimates.

In the other two categories—participants living in outbreak areas but without reported case-contact exposure and general populations in areas without known cases of EVD—exposure was either not well characterised or not well known. Even where EVD cases had not been reported, zoonotic exposure or different forms of disease manifestation could not be ruled out. The highly heterogeneous nature of these study populations makes any single summary estimate inappropriate. In outbreak areas estimates ranged from 0.9 to 17%, and in general populations described as unexposed estimates ranged from 0 to 24%.

### Evidence of assay validation

Few teams reported any validation of the assays used. Some studies repeated analyses with the same technique, usually in a US or European laboratory, but only seven of the 51 studies reported validation work through a different diagnostic platform. Of these, two retested a proportion of IFA positives against ELISA, finding close to 100% consensus^26,30^. Three tested ELISA against western blot of which two found 100% specificity^38,46,53^; the third did not report results^41^. Another found 77 and 75% specificity for ELISA against western Blot and IFA respectively^34^, and a further study confirmed IFA results by western blot but did not report results^33^.

Two studies in Sierra Leone included field testing of ELISA assays in PCR-confirmed positive samples from EVD survivors and community controls with no known exposure to EVD cases from the research area. One, using a novel IgG-capture ELISA^54^, found 95.9% (95%CI 89.9–98.9%) sensitivity and 100% specificity (95%CI 98.9–100%) using oral fluid samples from 97 survivors and 339 community controls^51^. The other, using the commercially available ALPHA Diagnostics assay, selected a cut-off that gave 96.7% sensitivity and 97.7% specificity in serum samples from 30 survivors and 132 community controls^50^.

## Discussion

We identified 51 studies covering 84 sample populations of 44,147 subjects reported to have had no symptoms of EVD during the outbreak period or to come from populations with no known outbreaks. Most data originated from Western and Middle Africa, and were collected during epidemiological investigations around outbreaks, or in serosurveys in countries without outbreaks but with similar ecology and animal hosts, which aimed to map the geographical extent of the virus. Some studies reported retrospective analysis of samples collected for other reasons prior to the first known outbreak in 1976.

An important finding of our review is the extreme heterogeneity of the studied populations and the lack of clarity in describing their exposure levels. We found that while some studies characterised their sample population clearly by level of contact and presence of symptoms, in many the level of contact/exposure was less clear, and some did not separate results for symptomatic and asymptomatic subjects. This makes comparison of results difficult, and combining results from the majority of the studies impossible. It may also explain the wide variation of findings which have perplexed investigators over time.

Many studies also employed very different cut-offs to define seropositivity meaning a simple review of results can be misleading. For our analysis, we excluded any study that used a cut off below≥1:64 for the studies using IFA, based on the advice in the literature, but there is no definitive evidence that this is an appropriate threshold. The cause of low IFA titre and whether it reflects false positives, or waning antibody response resulting from historical infection which may or may not have been symptomatic, has been frequently discussed. Recently 10 of 12 survivors from Yambuku were reported to have varying degrees of EBOV GP and NP reactivity by ELISA, 40 years after the outbreak^55^. Other studies have shown positive ELISA results in survivors up to 11 years after infection, but neither reported IFA results for comparison^56^.

There is no international reference measurement procedure for ebolavirus antibodies and the World Health Organisation has acknowledged the urgent need for one. Interestingly, given the scepticism often expressed regarding the specificity of IFA techniques in ebolavirus serology, a WHO collaborative study undertaken in 2015 to identify an interim reference standard found IFA no less specific or sensitive than the other methods employed, but only a few samples were tested^57^.

There are several limitations to the work presented here. The full information necessary for precision or clear interpretation was often not available. To pursue as high quality research as possible, we have focussed on publications that have undergone peer review and did not search grey literature. With the exception of Kuhn *et al.*^52^, which has been the standard reference on filovirus seroprevalence surveys to date, we did not search books. In addition to the limitations of the studies themselves noted above and in Table 1, we also note that the distinction of symptomatic and asymptomatic in the papers relied on self-reported health status, which may not be reliable.

To conclude, we present here a comprehensive updated review of seroprevalence surveys for ebolavirus infection in order to better understand the variation in rates found. We highlight the urgent need for validated standardised assays and for detailed characterisation of study population exposures to enable more generalizable estimates of the extent of asymptomatic ebolavirus infection to be made.

## Methods

### Search strategy and systematic review

A systematic search was done in PubMed to identify peer-reviewed papers presenting original data on ebolavirus infection seroprevalence using the following search string:

ebola AND (asymptom* OR antibod* OR IgG OR immun* OR ELISA OR serol*) NOT vacc* NOT immuniz* AND (Humans[Mesh])

No limitations were placed on language or location of study. Reference lists of the most comprehensive review to date^52^ and other papers were also reviewed. Although the focus of interest was data on subjects reported not to have symptoms at the time of an outbreak, we included papers reporting seroprevalence in all populations apart from those with diagnosed EVD in the initial review to ensure relevant studies were not missed.

The search produced 355 citations which were reviewed by title and abstract. Inclusion criteria were: investigation of any African species of ebolavirus immunoglobulin G (ie. not Reston) in individuals without ebolavirus symptoms or in general population groups, with information on denominators and seropositivity and description of those tested. The same search but limited to 2008 to 2016 was rerun on Web of Science; references prior to 2008 were checked against Kuhn *et al*’s list^52^. Four additional citations were found on Web of Science but none were retained for detailed reading. Six citations for papers not already included were identified from reference lists and retained for detailed reading.

Total citations: 365 of which 297 (81%) discarded for the following reasons:

- Detailed immunology or genetics with no relevant data collection for seroprevalence
- Description of acute phase diagnosis and/or investigation of convalescent subjects
- Epidemiology and/or treatment of symptomatic confirmed cases without investigation of non-case populations
- Investigations on sample populations without identifiable non-symptomatic individuals
- Studies examining immune response related to vaccination trials
- Review/comment articles without original data
- Modelling papers without original data
- Preliminary or duplicate reports of the same research study/data.

Sixty-eight papers were read in detail after which a further 20 were discarded for the reasons above. Data extracted from the remaining 48 papers included date of sera collection, composition of study population(s) in terms of exposure, location, selection process and any other defining characteristics, assay type, technique and antigens used, positivity threshold, number of participants per population type, number/proportion of IgG positive individuals, and any information on repeatability or test validity. All selected papers were scrutinised by both authors independently and results discussed and reconciled.

The last search was made on 31 July 2016. Two presentations from the 2016 Conference on Retroviruses and Opportunistic Infections (CROI, Feb. 2016) and one from the 8th International Symposium on Filoviruses (Sept. 2016) describing findings from the 2014–2016 outbreak were also included. A paper reporting one of the CROI presentations has subsequently been published (Nov 2016) and is referenced.

### Categorisation of exposure

Many of the studies reported results on sub-populations with different exposures. To reduce heterogeneity for analysis we categorised these sub-populations under three broad headings according to the extent of exposure: household or known case-contact; living in outbreak areas but without reported case-contact; and subjects drawn from general populations in locations without known EVD. Where study populations were reported to include symptomatic cases and gave enough information to identify these cases, we removed them and recalculated results.

We excluded one study of PCR negative ‘suspects’ with close, no or unknown contact exposure due to lack of information on symptom status^58^. In two other studies, sub-groups were not included in the table because they were reported to include symptomatic cases but gave insufficient information to allow recalculation of the seroprevalence estimate excluding those with symptoms^1,18^.

### Interpretation of seropositivity

We have recorded seropositivity results by antigen species where reported; where results were not reported by species, we record positivity to ‘ebolaviruses’. ‘Overall’ positivity is noted where it was reported or where it was possible to rule out double-counting.

To expose the problem of the different positivity thresholds used, we have recorded all studies and their reported cut-off in Table 1. Study characteristics and results have also been formatted as a machine-readable open access dataset (Data Citation 1).

### Data visualisation

**Figure 1:**
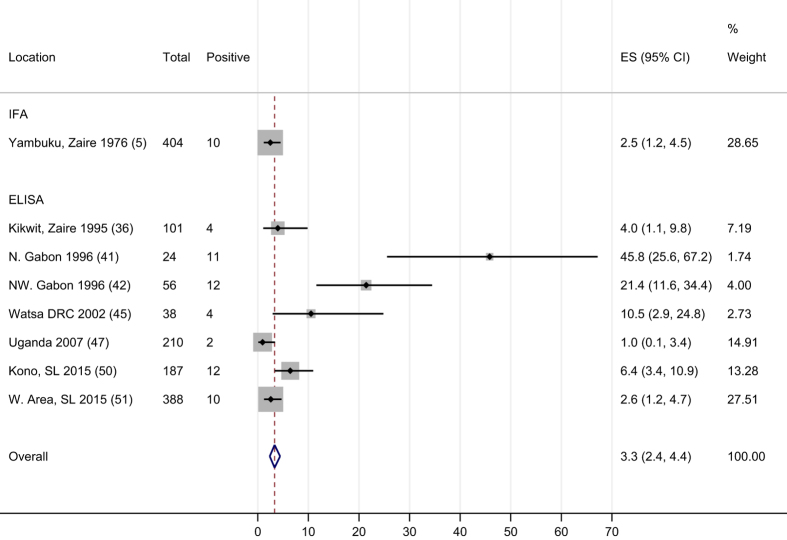
Forest plot and meta-analysis of seroprevalence of ebolavirus IgG among contacts of EVD cases reported to be asymptomatic during the outbreak period. Further details of each included study are given in Table 1. Legend: Ref: reference number; IFA: Immunofluorescence Assay; ELISA: Enzyme-linked immunosorbent assay; ES: Estimated proportion; N, NW: North, Northwestern; SL: Sierra Leone; W. Area: Western Area Province. Note: Zaire now Democratic Republic of Congo; Rhodesia now Zimbabwe.

**Figure 2:**
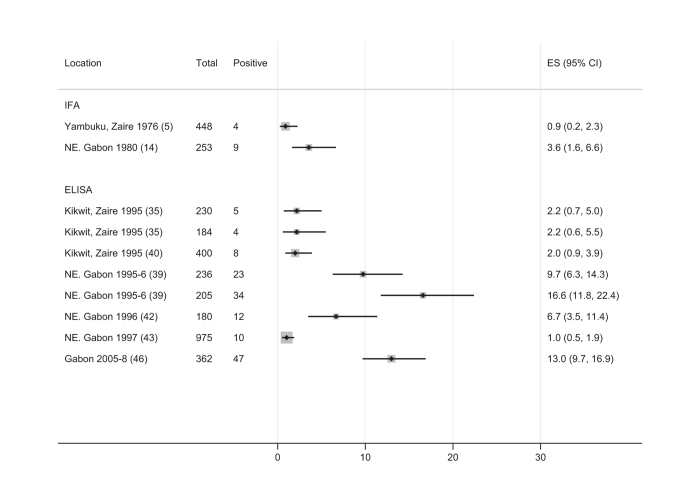
Forest plot of seroprevalence of ebolavirus IgG in individuals reported to be asymptomatic during the outbreak period, recruited in areas with known EVD cases, excluding direct contacts of EVD cases. Further details of each included study are given in Table 1. Legend: Ref: reference number; ES: Estimated proportion; IFA: Immunofluorescence Assay ELISA: Enzyme-linked immunosorbent assay; DRC: Democratic Republic of Congo; N, NE: North, Northeastern.

**Figure 3:**
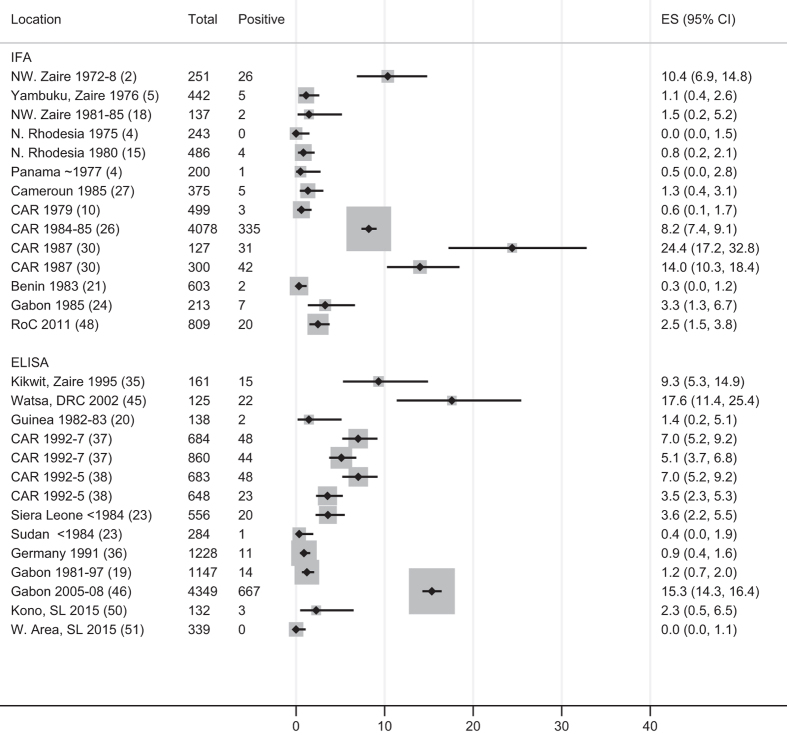
Forest plot of seroprevalence of ebolavirus IgG in general populations living in areas without reported EVD cases. Further details of each included study are given in Table 1. Legend: Ref: reference number; IFA: Immunofluorescence Assay; ELISA: Enzyme-linked immunosorbent assay; ES: Estimated proportion; IFA: Immunofluorescence Assay; ELISA: Enzyme-linked immunosorbent assay; DRC: Democratic Republic of Congo; RoC: Republic of Congo; CAR: Central African Republic; N, NW: North, Northwestern. Note: Zaire now Democratic Republic of Congo; Rhodesia now Zimbabwe.

To summarise the data visually and present 95% confidence intervals, we created Forest plots for each of the three exposure categories (Figs 1,2,3) which allow results to be compared in the different contact groups. To address the problem of varying thresholds, we included only those IFA studies that reported results according to the 1:64 titre cut-off cited as more stringent by WHO and others^5,18,21,59^, or which reported enough detail for this threshold to be applied. For ELISA studies, the range of methods used to define positivity was too wide to assign a common threshold so all have been included in the Forest plots, with their method of defining the cut-off detailed in Table 1 (available online only).

### Statistical analyses

We performed a meta-analysis using the Freeman Tukey arcsine square root transformation method and ‘fixed effects’ (weighted average) inverse variance (*metaprop*, STATA^60^) on the eight study populations with known-case contact. We chose a ‘fixed effects’ (weighted average) model as contact should give similar risks in different contexts, and because random effects models give too much weight to small studies^61^. We present an pooled summary estimate for the group with known contact exposure (Fig. 1). We do not show summary estimates for the groups covering subjects living in outbreak areas but without reported case-contact, or drawn from general populations in locations without known EVD (Figs 2 and 3) as these populations are likely to have very different exposure levels so an overall summary estimate of prevalence would be meaningless.

## Additional information

**How to cite this article**: Bower, H. & Glynn, J. R. A systematic review and meta-analysis of seroprevalence surveys of ebolavirus infection. *Sci. Data* 4:160133 doi: 10.1038/sdata.2016.133 (2017).

**Publisher**’**s note**: Springer Nature remains neutral with regard to jurisdictional claims in published maps and institutional affiliations.

## Figures and Tables

**Table 1:** Findings of seroprevalence studies investigating presence of ebolavirus immunoglobulin G antibodies in ‘asymptomatic’ populations, 1961–2016

**Location**	**Year sera collected**	**Population type (as described in paper)**	**Group**	**No. of samples**	**# IgG +ve**	**Species % +ve**	**Assay type**	**Cut-off (as described in paper**	**Antigen & validation information**	**Notes**
Assab, Awash, Blue Nile, Illubor, & Ogaden regions, Ethiopia^1^	1961/62	Asymptomatic individuals from area not affected by ongoing Yellow Fever epidemic	C	178	42	EBOV 24%	IFA(w)	≥1:16	Antigens: Polyvalent ELM (Ebola-Lassa-Marburg viruses) & monovalent EBOV (Mayinga): all sera reacting with ELM also reacted with monovalent EBOV antigen but not with monovalent Marburg and Lassa.	Samples from symptomatic Yellow Fever-negative individuals also tested: 12% positive.
N.W. Zaire (now Democratic Republic of Congo DRC)^2^	1972–78	General population from area west of Yambuku (site of 1st recorded outbreak in 1976)	C	251	4326	EBOV 17.1%10.4%	IFA(w)	≥1:16≥1:64	Antigen: EBOV (May. 80826)	
Harbel, Bong town, Yekepa, Liberia^3^	1973	Staff & family members of rubber and mining companies	C	592	83	Ebolavirus 14.0%	ELISA/WB	Not stated	Antigen not stated	Sera taken in 1973 and investigated for Lassa and ebolavirus in ~1986. Ebola statistic reported without further information.
Northern Rhodesia (now Zimbabwe)^4^	1975	‘Control group’	C	243	20	EBOV 0.8%0.0%	IFA(w)	≥1:8≥1:64	Antigen not specified in report: Kuhn^52^ notes antigen as EBOV	Areas sampled had no known outbreak
Yambuku, Zaire (DRC)^5^	1976	Asymptomatic contacts of cases	A	404	10	EBOV 2.5%	IFA(w)	≥1:64	Antigen: EBOVValidation: Repeat testing of the 4 antibody positive with no known contact gave the same results. 32/33 (97%) positive samples (including samples from cases) confirmed positive by CDC (US)^4^ (p141)	
		Residents from villages with cases but no known contact	B	448	4	EBOV 0.9				
		Residents from 4 neighbouring villages with no cases	C	442	5	EBOV 1.1%				
Maridi, Sudan (now South Sudan)^6^	1976	Close family contacts	A	93	13	SUDV 14.0%	IFA(w)	≥1:8	Antigen: SUDVValidation: 42 of 48 clinically diagnosed survivors from Nzara (87%) were considered positive using the same IFA protocol. Several samples from Nzara were retested and confirmed positive by CDC (US) using the same protocol.	9 antibody positive family contacts had symptoms and have been excluded from these figures. Not clear if all subjects in this group were interviewed about symptoms.
		Asymptomatic Maridi schoolboys with no known contact	B	29	3	SUDV 10.3%				
		Asymptomatic hospital staff with probable/possible contact	A	64	7	SUDV 10.9%				4 nurses; 1 cleaner, 1 toilet cleaner, 1 water carrier were positive
Nzara, Sudan (now South Sudan)^6^	1976	Asymptomatic cotton factory workers (site of index case but reportedly no direct contact)	B	109	7	SUDV 6.4%	IFA(w)	≥1:8		Among factory workers titre range was 1:16–1:32
		Close family contacts of clinically diagnosed cases	A	78	1	SUDV 1.3%				Only 6/31 (19.4%) of clinically diagnosed subjects were antibody positive, and none had levels>1:32
San Blas Islands, Panama^4^	1977	San Blas Indians	C	200	1	EBOV 0.5%	IFA(w)	≥1:64	Antigen: not specified in report: Kuhn^52^ records antigen as EBOV	Areas sampled had no known outbreak. Method of selection not known
Tandala, Zaire (DRC)^7^	1977–78	Missionaries and ‘a few’ hospital staff with case contact (1977)	A	50	0	EBOV 0%	IFA(w)	≥1:16	Antigen: EBOV	One doctor, who tested positive in 1977 and 1978 and had history of severe illness after attending the autopsy of a haemorrhagic fever victim in 1972, is excluded. Some individuals gave samples in both the 1977 and 1978 groups
		Hospital staff with case contact (1978)	A	71	0	EBOV 0%				
		Residents of villages with confirmed /suspect cases	B	346	21	EBOV 6.1%				
		Residents of other villages in same area	B	750	58	EBOV 7.7%				
Liberia ^8^	1978-79	Random rural general population in multiple counties	C	433	26	Ebolavirus 6.0%	IFA(w)	≥1:16	Antigens: unspecified ebolavirus, Marburg & Lassa viruses. No sera positive for ebolavirus was positive for MARV or LASV.	No known outbreak. Titre range: 1:16 to 1:1024
Nzara/Yambio, (South) Sudan^9^	1979	Asymptomatic adult family members of cases with known physical contact	A	38	12	Ebolavirus 32%	IFA(w)	≥1:16	Antigen: unspecified	
		Asymptomatic adult family members of cases who denied physical contact	B	23	3	Ebolavirus 13%				
		Adults from families without known cases in same area	B	45	8	Ebolavirus 18%				Unknown if these people were exposed in 1976 outbreak, which could explain the high prevalence
Bangassou, Central African Republic (CAR)^10^	1979	General population in forest and semi-forest zones	C	499	103	Ebolavirus 2.0%0.6%	IFA(w)	≥1:16≥1:64	Antigen: Polyvalent of unspecified ebolavirus, MARV & LASV, followed by monovalent test for positive samples. Positive sera sent to CDC (US) for repeat testing; results not reported.	Areas sampled had no known outbreak
Moloundou, Lolodorf Bipindi, Lomie, Yaounde & Pete, Cameroon^11^	1980	General population in five regions (forest, pre Sahelian savannah and the capital) and different ethnic groups	C	1517	147	Ebolavirus 9.7%	IFA(w)	≥1:16	Antigens: unspecified ebolavirus provided by CDC (US)	No known outbreak. Positives in all areas, range 3%-23%. Highest in Pygmies and rain forest farmers. 6% in the capital, Yaoundé. Report to OCEAC in the same year gave positivity of 6.2% (51/821) in Moloundou, compared to 13.2% for the same location in this study, and 29% (20/70) in Mbatika, but positivity threshold used is not reported.^63^
Lugulu, western Kenya^12^	1980	Family and close neighbours of an IFA confirmed case (asymptomatic?)	A	84	4	Ebolavirus 4.8%	IFA (?)	≥1:16	Antigens: monospecific, triple (unspecified ebolavirus, MARV, LASV) and poly-antigen (CCHFV, RVFV, ebolavirus, LASV, MARV).Validation: sera examined at National Institute of Virology, Johannesburg and CDC(US): labs used different thresholds, so positive confirmed only where both found ≥1:16	Area in Western Kenya, close to Nzoia. Samples collected during investigation of 2 MARV suspect cases who were later shown to be ebolavirus positive.
Kenya^13^	1980	Different studies in 5 regions of Kenya:—Lodwar, Laisamis, Masia, Malindi/Kilifi- Nzoia	C	1058841	189	EBOV/SUDV 1.7%1.1%	IFA(w)	≥1:16	Antigens: inactivated unspecified ebolavirus, MARV, CCHFV, RVFV, & LASV; positives tested against EBOV(May) & SUDV (Boniface & Maleo). Authors report ‘most’ of the Nzoia samples were only tested against EBOV(May)	No known outbreak but Nzoia cohort reported to include suspected cases and their contacts. Highest prevalence: Lodwar 7.8% (north-west Kenya). Note referenced paper includes some sera reported on in other papers.^72^
Haute Ogooue, Gabon^14^	1980	General population in outbreak area but no known contact	B	253	16851218	EBOV 6.3%3.2%SUDV 2.0%0.4%EBOV/SUDV 8.3%3.1%	IFA(w)	≥1:16≥1:64≥1:16≥1:64≥1:16≥1:64	Antigens: inactivated polyvalent unspecified ebolavirus/LASV MARV: positives tested against EBOV(802850) & SUDV(802681).	Samples from the Occupational Health Services, plus 28 women & their newborns. One sample was positive ≥1:64 on both EBOV & SUDV
Northern Rhodesia (now Zimbabwe)^15^	1980	Asymptomatic schoolboys (8-10y): no known outbreak	C	486	94	EBOV 1.9%0.8%	IFA(g)	≥1:8≥1:128	Antigen: polyvalent CCHFV, RVFV, LASV, MARV, unspecified ebolavirus slides provided by CDC (US): positives tested against individual antigens (EBOV & SUDV)Validation: 4 of 5 positive samples sent to CDC (US) were ≥1:128 in repeat IFA testing.	None were positive for SUDV.
Pool, Congo-Brazzaville (now Republic of Congo)^16^	1981	Children from 20 villages aged 3-15 years and unvaccinated for smallpox	C	790	119	Ebolavirus 15.0%	IFA(?)	Not stated	Antigen: polyvalent CCHFV, RVFV, LASV, MARV, unspecified ebolavirus; also tested against monovalent antigens	Pool region is on the border with DRC. Areas sampled had no known outbreak but populations were selected for close contact with animals
Grand Bassa, Liberia^17^	1981-82	Individuals asymptomatic for EVD consisting of 106 epilepsy patients; 87 healthy relatives of these patients; 32 unrelated geographically matched controls.	C	225	26429	EBOV 11.6%SUDV 1.8%Overall 12.9%	IFA(w)	unclear	Antigen: polyvalent CCHFV, RVFV, LASV, MARV, EBOV (May), SUDV(Bon) (known as CRE_2_LM); positives retested against individual antigens.Validation: Difficulties with non-specific binding led researchers to replicate and use blinded observers to read results. Only samples with unequivocally positive results by 2 observers were considered positive.	No known outbreak. Similar proportion positive in each participant group. 38% epilepsy patients had a febrile illness 1-4 weeks before onset of epilepsy, but no significant difference in seroprevalence with & without febrile history. Paper says 30 positives in total but one counted twice (positive for EBOV and SUDV). Titres ranged from 32-128 for EBOV; 6/29 had antibodies to more than 1 virus.
Sud-Ubangi sub-region, DRC (includes Tandala)^18^	1981-85	Age/sex matched controls from the same villages as reported cases	C	137	2	Ebolavirus 1.5%	IFA(w)	≥1:64	Antigen: polyvalent CRE_2_LM; positives tested against unspecified ebolavirus-specific antigens.	In addition 188 contacts of possible and probable cases were tested; 28 were positive at ≥1:64 but all had had symptoms fitting the definition of a possible or clinical case. It is not clear how many of the other contacts had symptoms.
Northeastern, southeastern & western Gabon^19^	1981-1997	Six rural communities (Makokou, Doussala, Doussieousou, Matadi-Ngoussa, Moukoro, Latoursville): sera gathered during onchocerciasis research	C	1147	14	EBOV 1.2%	ELISA (k)	Mean +3SD of OD of negative controls	Antigen: EBOVValidation: In 2003, 6 of original 14 positives were re-bled (others unavailable): 2 were still positive. 14 controls (relatives and ‘cohorts’) were unreactive	6 seropositives were from north-eastern Gabon where outbreaks had occurred; 8 were from western communities more than 500km from known epidemics. Authors also investigate and correlate animal with human outbreaks. Conclude that less virulent strains of EBOV affected western areas.
Madina-Ula, Guinea^20^	1982-83	Healthy adults sampled during an outbreak of an unknown disease	C	138	1142	EBOV 7.8%2.9%2.2%	ELISA(r)ELISA(r)IFA(b)ELISA(r)IFA (b)	≥1:8≥1:512≥1:16≥1:512≥1:64	Antigen: EBOV	Areas sampled had no known outbreak
Benin^21^	1983	General population, non-outbreak country	C	603	2	EBOV or SUDV? 0.3%	IFA (?)	≥1:64	Antigen: EBOV, SUDV	Unpublished data cited by Gonzalez *et al* (2005): no further information, not specified which ebolavirus antigen samples were reactive to.
Ethiopia, Awash valley^1^	1983	Unexposed children	C	250	0	EBOV 0.0%	IFA(w)	≥1:16	Antigens: Polyvalent ELM (Ebola-Lassa-Marburg viruses) & monovalent EBOV (May)	Areas sampled had no known outbreak
Karamoja, Uganda^22^	1984	‘Healthy’ adults 20-40y recruited during visits to a health centre, excluding any with current or recent fever	C	132	44	EBOV 3.0%SUDV 3.0%	IFA(w)	unclear	Antigen: polyvalent CCHFV, RVFV, LASV, MARV, EBOV (May), SUDV(Bon) (CRE_2_LM); positives retested against individual antigens.	No known outbreak. Not clear if some samples had antibodies to both EBOV & SUDV. Not specified how many>1:64. All samples<1: 128.
Mobai, Sierra Leone & unspecified location Sudan^23^	<1984	No information on population type: general population assumed from description- Mobai, Sierra Leone- Sudan	C	556284	101010	EBOV (a) 1.8%(b) 1.8%(a) 0.35(b) 0	(a) ELISA +ve/IFA+ve (b) ELISA +ve/IFA −ve or unclear	ELISA: +ve within 2SD of +ve ref. sera; −ve within 1SD of negative ref seraIFA≥1:100	Antigen: EBOVValidation: All? samples were tested using both assays	Year of sample collection is not recorded. Paper reports a high level of cross reactivity with MARV, lasting a number a number of years after infection.
Haute Ogooue, Gabon^24^	1985	Inhabitants of Ambinda village	C	213	20621227	EBOV 9.4%1.4%SUDV 0.9%0.5%Overall 10.3%3.3%	IFA (j)	≥1:16≥1:64≥1:16≥1:64≥1:16≥1:64	Antigen: polyvalent CCHFV (IB-AR-10200 Nigeria), RVFV (ZH-501 Egypt), LASV (Josiah), MARV (Musoki), EBOV (May), SUDV(Bon) (CRE_2_LM); positives retested against individual antigens.	No known outbreak
Nola,Ikaumba, Bozo, Bangassou, Mbre & Birao, Central African Republic ^25^	1984-85	General population from 5 ecological regions including one close to Zaire/DRC outbreak area	C	836	15263	EBOV 18.2%SUDV 7.5%	IFA(j)	≥1:16	Antigens: EBOV (May), SUDV (Bon), MARV (Mus)	No known outbreak but Zemio which borders DRC accounted for 43% of EBOV positives
Nola,Ikaumba, Bozo, Bangassou, Mbre & Birao, Central African Republic ^26^	1984-85	Asymptomatic general population from 5 ecological distinct zones selected on accessibility: additional villages wer chosen where multiple ethnic groups coexisted.	C	4295*4078*	681209853259914335	EBOV15.9%5.1%SUDV 19.8%6.4%Overall 21.3%8.2%	IFA (j)	≥1:16≥1:128≥1:16≥1:128≥1:16≥1:128	Antigens: polyvalent EBOV (May), SUDV (Bon), MARV (Mus), LASV (Jos), CCHFV (10200), RVFV(ZH501); positives (≥1:16) retested against monovalent antigen.Validation: 185 samples were reanalysed by ELISA in 1996: results confirmed original analysis.^21^	Study linked to one above in CAR^25^ but using different set of sera sampled in the same period.* Two different denominators are cited: 4296 people are reported for all titre levels; 4078 people are reported in the table describing only samples showing titres≥1:128.Highest prevalence in woods and forest regions. 86% of titres≥1:64 but reached as high as 1:2048
Nkongsamba, Cameroon^27^	1985	Randomly selected urban general population (15-44 years)	C	375	75	Ebolavirus 1.9%1.3%	IFA (?)	≥1:16≥1:64	Antigens: polyvalent unspecified ebolavirus, CCHRV, RVFV, MARV)	These samples were included in the following multi-country study which used a different threshold.^28^ One sample was positive for both ebolavirus and RVFV
Central Africa^28^ (now Middle Africa)	1985-87	Randomly selected sera collected in:Cameroon (Mora, Maroua, Nkongsamba)Central African Republic (Bangui)Chad (N’djamena)Republic of Congo (Pointe Noire, Brazzaville)Equatorial Guinea (Bioco Island, Nsork)Gabon (Libreville, Port-Gentil, Ogooue-Ivindo, Haut Ogooue, Ngounie)	C	11523273347286881841	8910733451111259	EBOV/SUDVλ7.7%32.7%3.6%7.0%16.1%14.0%	IFA (w)	≥1:16	Antigens: polyvalent EBOV (May), SUDV (Bon), MARV (Mus), LASV (Jos), CCHFV (10200), RVFV(ZH501); positives (≥1:16) retested against monovalent antigen.	Areas sampled had no known outbreak
Chobe, Northern Botswana^29^	1984-86	1984: 52 asymptomatic villagers)1985: 25 villagers with non-specific or ictero-haemorrhagic symptoms1986: 77 asymptomatic villagers	C	154	0	EBOV/SUDV 0%	IFA (J)	≥1:16	Antigens: polyvalent CCHFV, RVFV, LASV, MARV, EBOV (May), SUDV(Bon): positives re-tested against monovalent antigens.	Areas sampled had no known outbreak. Unable to separate results for the symptomatic group. Only reaction found was against RVFV. Testing performed in Paris.
Lobaye, Central African Republic^30^	1987	Asymptomatic general population, Lobaye district: Pygmy hunter-gathers	C	127	31	EBOV/SUDV 24.4%	IFA (J)	≥1:128	Antigens: polyvalent EBOV (May), SUDV (Bon), MARV (Mus), LASV (Jos), CCHFV (10200), RVFV(ZH501); positives (≥1:16) retested against monovalent antigen, considered reactive if≥1:128.Validation: 296 samples from this study and 185 samples from the CAR 1984-85 study above were re-analysed in 1996 using ELISA (≥1:400 & sum of 4 ODs≥1.000). 6.2% were Ebola IgG positive (30/481) compared to 6.4% in these samples previously by IFA.^21,26^	Area with no known outbreak.Of the positives, 45 reacted to both EBOV & SUDV: it is not possible to identify how this splits between the groups.
		Asymptomatic general population Lobaye district: Mozombo/Mbati subsistence farmers	C	300	42	EBOV/SUDV 14.4%				
Nigeria^31^	1988	Asymptomatic general population in different locations	C	1677	3022	SUDV 1.8%EBOV/SUDV 1.3%	IFA(w)	≥1:10	Antigens: polyvalent CCHFV, RVFV, LASV, MARV, EBOV (May), SUDV(Bon): positives with titre≥1:10 retested against monovalent antigens. Known positive/negative controls used	Areas sampled had no known outbreak. All positive samples came from savannah areas (Benue/Gongola)Of the positives, none reacted to EBOV alone.
Antanarivo, Mandoto, andasibe, Tsiroanomandidy & Ampijoroa, Madagascar^32^	1989	Asymptomatic adults from 5 different areas (urban & rural, cattle-lands, forested)	C	381	17	EBOV 4.5%	IFA (j)	≥1:16	Antigens: polyvalent CCHFV, RVFV, LASV, MARV, EBOV (May), SUDV(Bon): positives retested against monovalent antigens	Areas sampled had no known outbreak. Range of titres: 1:16 to 1:512; highest prevalence in the capital Antanarivo 13.3%.
United States^33^	1990	CDC (US) employees with current or previous occupational exposure to monkeys. None ill.	B	550	42	EBOV/SUDV/RESTV/MARV 7.6%	IFA (?)	≥1:16	Antigens: EBOV, SUDV, Reston ebolavirus (RESTV), MARV.Validation: confirmed by western Blot	This paper summarises 2 others ^73,74^Results are for positivity to at least one of the four antigens, which include Marburg.
		Adult primary care outpatients in US	C	449	12	2.7%				
Germany^34^	1991	Various groups of healthy individuals, blood donors and routine diagnostic samples, plus 56 individuals who had had contact with Marburg patients in 1972.	C	1288	1144	EBOV 0.85%RESTV 3.4%	ELISAIFA or WB	ELISA: 1:100IFA: 1:40WB: +ve if stained≥2 viral proteins)	Antigens: EBOV (May), RESTV, Marv(Mus)Validation: Considered positive if ELISA confirmed by IFA or WB.Confirmation: ELISA vs IFA 75%; ELISA vs Western Blot 77%.	Authors state that the sample groups showed no significant differences in the prevalence of antibody against the 3 filoviruses and so they treated as one group for analysis and only overall results reported.WB results: ‘most’ sera reacted with the NP protein, ‘less’ with VP40, VP35 & VP30, and ‘few’ with VP24. None reacted to GP or L proteins.
Kikwit, DRC^35^	1995	Four forest site populations near Kikwit town, site of outbreak	B	230	5	EBOV 2.2%	ELISA (k)	≥1:400 & OD sum≥1.25	Antigen: unspecified but Kuhn^52^ reports EBOV; sera tested in CDC (US) Special Pathogens Lab.	Differentiation between forest and city workers was difficult: publicity brought people out of their areas: self identified occupations. 95% of participants including all 9 positives said they knew someone with Ebola.
		City workers, Kikwit	B	184	4	EBOV 2.2%				
		Asymptomatic volunteers from unaffected villages near Kikwit	C	161	15	EBOV 9.3%				5/15 positives knew someone who had had Ebola
Kikwit, DRC^36^	1995	Household contacts aged 3 m-58y	A	101	4	Ebolavirus 4.0%	ELISA (k)	≥1:400 & OD sum≥1.25	Antigen: unspecified but probably EBOV; sera tested in CDC (US) Special Pathogens Lab.	Paper cites 5 positive sera but 1 miscarried 3 days before giving her positive specimen so fits case definition for Ebola. One of the remaining 4 may have acquired Ebola by sexual transmission from a convalescent. Out of 81 sero-negative household contacts, 15 had episodes of illness fitting case definition at some point during follow-up.
Central African Republic ^37^	1992-97	Pygmy general population: southern regions of CAR (Lobaye, Belemboke)	C	684	48	EBOV 7.0%	ELISA (n)	≥1: 400	Antigens: EBOV, MARV, RVFV, LASV, Yellow fever (YF) Hantaviruses (Seoul, Puumala and Thottapalayam)Validation: 244 sera taken in Lobaye in 1995 (11.6% ELISA positive to EBOV) were retested with IFA to EBOV (May) & SUDV (Bon): 34% were positive.	Prevalence of EBOV seropositivity varied between 2% and 13% in different participant groups.
		Bantu villagers: southern region of CAR (Lobaye, Belemboke, Nola, Bangassou)	C	860	44	EBOV 5.1%				
Central African Republic^38^	1992-95	Pygmy subgroup (Lobaye, Belemboke: all sites no known outbreaks)	C	683	48	EBOV 7.0%	ELISA (k)	Mean+2SD of negative controls ≥1:400 & OD sum of 4 dilutions>1.0	Antigens: EBOV (May) Marv(Mus); tests performed by Institut Pasteur, Bangui.Validation: 14 positive & 54 negative samples sent to CDC (US) to be tested against strain antigens: all results confirmed.	Primary or secondary forest areas with some agricultural activities
		Non-pygmy subgroup (Lobaye, Belemboke, Bangassou, Nola: all sites no known outbreaks)	C	648	23	EBOV 3.5%				
Ogooue Ivindo, Gabon^39^	1995-96	Residents of 3 encampments (Andock, Minkebe, Mekoua) in the area where the epidemic occurred (including some contacts)	B	236	23	EBOV/SUDV/RESTV 9.7%	ELISA (k)	mean+3SD of negative controls	Antigens: EBOV, SUDV, RESTV with known positive/negative controls	1 positive serum from a survivor excluded from encampment group; unclear how many known case contacts are included in this group.
		Residents of 3 outbreak villages (Mayibout 1 & 2, Mvadi) where cases were reported during the outbreak	B	205	34	EBOV/SUDV/RESTV 16.6%				
Kikwit^40^	1995	Healthcare workers in outbreak area (70% hospital; 30% health centre) who did not have known EVD	B	400	8	EBOV 2.0%	ELISA (k)	Sum of adjusted OD >1.25	Antigen: EBOV	The 8 positives were from a group of 12 samples which were ‘borderline positive’ on 1st test. Only 4 of these samples were retested: all were negative and have been excluded.129 of the 402 subjects reported being ill during Ebola period. Two with fever and haemorrhage (tested EBOV negative) have been excluded.
Gabon ^41^	1996	Selected asymptomatic family members directly exposed to body fluids during outbreaks in 1996	A	24	11	EBOV 45.9%	ELISA (k)	Mean adjusted OD for 10 control samples	Antigen: EBOVValidation: confirmed with western blot on NP and VP40 proteins	Subjects were asymptomatic throughout and were sampled several times. 1st samples showed no antibodies suggesting no prior immunity; IgG appeared 15-18 days after first possible exposure.Paper also describes results of viral RNA detection after 2 rounds of RT-PCR, finding positive results in 7/11 antibody-positive individuals tested and 0/13 antibody-negative individuals.
Nouna River, Ogooue-Ivindo, Gabon^42^	1996	Residents in gold-mining villages with contact exposure in 1995 epidemic	A	56	12	EBOV 21.4%	ELISA (?)	OD>mean +2SD of 3 known negative controls	Antigen: ebolavirus Gabon 95-39/3 (Centre International de Recherches Medicales de Franceville)	All subjects reported fever and diarrhoea at least once in 1-year period of study, but not haemorrhagic symptoms. IgG positive titre range (OD 310-2,666).Age, sex, ethnic group not associated with seropositivity. Non- significant difference in seropositivity in people on site during 1995 epidemic (8.2%) and not on site (3.7%), among those with no reported contact
		Residents in same villages without contact exposure	B	180	12	EBOV 6.7%				
Upper Ivindo River, Ogooue-Ivindo, Gabon^43^	1997	Individuals from 8 permanent villages in outbreak-prone region (4 survivors excluded)	B	975	10	EBOV 1.0%	ELISA (k)	Mean OD negative controls +3SD	Antigen: EBOV, performed in National Institute for Communicable Diseases South Africa.Validation: All positives plus a random selection of 28 negatives were retested with same protocol in CDC (US)—all were confirmed with response mainly directed to NP, VP40, VP35 and sGP viral proteins.	Serosurvey done in 1997; questionnaires done in 1999 on 10 positives: only 1 had contact, none were ill.
Belarus & Ukraine^44^	1997	‘Foreign visitors’ mostly from Africa: unclear if any had history of EVD symptoms	C	562	30	EBOV 5.3%	IFA (w)	Not specified	Antigens: EBOV (May), MARV (Voege) LASV (Jos)	Authors suggest positive results among foreign visitors reflect historic infection/ recovered cases, and unexpected results reflect cross-reactivity with infections such as malaria, HIV and influenza. Other observers suggest the results are just as likely to be artifact.^63^
		Belarus/Ukraine residents ‘at risk of HIV’	C	506	20	EBOV 4.0%				
		Blood donors from the Blood Transfusion Institute, MoH Belarus & workers at the Belorussian Scientific Research Institute of Epidemiology & Microbiology	C	131	21	EBOV 16.0%				
Watsa region, DRC^45^	2002	Efe tribe pygmies exposed to a possible case at some time in their lives in household, occupation or funeral setting; no history of haemorrhagic fever symptoms	A	38	4	EBOV10.5%	ELISA (k)	2×mean +3SD of negative controls value	Antigen: EBOVODs were expressed as percent positivity of a confirmed EBOV-positive sample; negative controls were from 60 South African subjects ‘almost certain’ to be seronegative.	A total of 300 people were sampled from 39 communities. 137 who reported experiencing haemorrhagic fever symptoms sometime in their life are excluded from this summary. 22% of those reporting symptoms were IgG positive.
		Efe pygmies no reported exposure to possible cases; no history of haemorrhagic fever symptoms	C	125	22	EBOV 17.6%				
Gabon^46^	2005-08	Random sample of asymptomatic people aged >16 years without exposure, over all 9 provinces of Gabon	C	4349	667	EBOV 15.3%	ELISA (k) & WB	Cut-off based on negative controls from a French population	Antigen: EBOV Validation: Random sample of 138 positives were tested by western blot in 2008 and all were positive to at least one EBOV antigen.^53^	Gabon experienced 7 outbreaks between 1994 & 2002 affecting >20 villages and towns; in total there were 208 cases and 151 deaths.
		Random sample of asymptomatic children from 6 villages in outbreak-prone province (Ogooue-Ivindo)	B	362	47	EBOV 12.9%				
Bundibugyo, Uganda^47^	2007	Adult contacts of survivors >18 y. Samples taken ~29 months after outbreak	A	210	2	EBOV/SUDV/TAFV/BDBV 1.0%	ELISA (s)	Mean OD of negative controls plus 3SD	Antigens: EBOV, SUDV, Tai Forest ebolavirus (TAFV), Bundibugyo ebolavirus (BDBV), MARV(Mus)Validation: 28/36 confirmed cases tested positive to BDBV, 20/36 to EBOV, 10/36 to SUDV, 12/36 to TAFV & 29/39 to any of the 4 strains.	15/223 contacts positive but 13 were symptomatic at the time of the outbreak, therefore only the 2 asymptomatic contacts are included in the table.
Republic of Congo^48^	2011	Healthy blood donors 18-65y, no known case exposure	C	809	20	EBOV 2.5%	Double IFA	Reciprocal endpoint titres ≥20	Antigens: EBOV (ATCC 1978), MARV (Popp 1967)Authors state: ‘double IFA’ technique has higher specificity than ‘regular’ IFA because only antibodies that detect filoviral antigens in co-localisation with a monoclonal antibody are considered.	Seropositivity ranged from 1.6–4% depending on city/rural location; 4% in Pointe Noire.
Liberia [PREVAIL]^49^	2015	Close contacts of cases. NB 126 of the contacts were sexual partners of survivors after discharge.	A	760	98	EBOV 12.9%	ELISA (Alpha)	unspecified	Antigen: EBOV	Preliminary results. Study excluded from meta-analysis of known case contact group (A) because unclear what proportion of participants were symptomatic.
Kono, Sierra Leone^50^	2015-16	Asymptomatic close contacts of cases aged≥4 years who had been resident in quarantined houses during the period of active Ebola transmission	A	185 s	12	EBOV 6.4%	ELISA (Alpha)	4.7 U/ml	Antigen: EBOV GPValidation: 29/30 PCR-confirmed EVD survivors, and 3/132 community controls were positive: 96.7% sensitivity, 97.7% specificity	2 other positives had fever. Not clear if negatives were asked about symptoms
		Individuals from 3 villages without reported cases	C	132	3	2.3				
Western Area, Sierra Leone^51^	2015	Household contacts of cases, asymptomatic at the time EVD was in the household	A	388	10	EBOV 2.6%	ELISA (PHE)	Mean OD of negative controls+fixed OD measure (0.1)	Antigen: EBOV GP. ‘Positive’ only if repeat test was positiveValidation: 93/97 PCR-confirmed EVD survivors and 0/339 community controls were positive: sensitivity 95.9% (95%CI 89.9–98.9%); specificity 100% (95%CI 98.9–100%)	Tests were done on oral fluid.
		Individuals from 3 villages in Western Area without reported EVD cases	C	339	0	EBOV 0%				
A=populations with household contact or known case contact										
B=populations without known contact but in outbreak areas or villages with cases										
C=general population—no known outbreak exposure or contact										
**Abbreviations:** CDC (US): Centers for Disease Control and Prevention, Atlanta, USA; PHE: Public Health England; EBOV: Zaire ebolavirus; SUDV: Sudan ebolavirus; BDBV: Bundibugyo ebolavirus; TAFV: Tai Forest Fever Virus; MARV: Marburg Fever Virus; CCHFV: Crimean Congo Haemorrhagic Fever Virus; RVFV: Rift Valley Fever Virus; LASV: Lassa Fever Virus; IFA: immunofluorescence assays; ELISA: Enzyme-linked immunosorbent assay; WB: Western Blot; OD: optical density; SD: standard deviation										
**Assay technique notation**										
IFA (w): Wulff & Lange^62^										
IFA( j): Johnson^63^										
IFA (g): Gardner^64^										
IFA (b) Baskirtsev^65^										
Double IFA: Emmerich^66^										
IFA (?) ELISA (?): technique not referenced										
ELISA (k): Ksiazek^67^										
ELISA (s): Schoepp^68^										
ELISA (v): Viral Haemorrhagic Fever Consortium (SL)^69^										
ELISA (n): Nicklasson^70^										
ELISA(r): Rezapkin^71^										
ELISA(PHE): Lambe^54^										
ELISA(Alpha): ADI^75^										
